# Habitat use and foraging parameters of breeding Skylarks indicate no seasonal decrease in food availability in heterogeneous farmland

**DOI:** 10.1002/ece3.8461

**Published:** 2022-01-26

**Authors:** Manuel Püttmanns, Laura Böttges, Tim Filla, Franziska Lehmann, Annika Sophie Martens, Friederike Siegel, Anna Sippel, Marlene von Bassi, Niko Balkenhol, Matthias Waltert, Eckhard Gottschalk

**Affiliations:** ^1^ Department of Conservation Biology Johann‐Friedrich‐Blumenbach Institute of Zoology and Anthropology University of Göttingen Göttingen Germany; ^2^ Institute for Medical Biometry and Bioinformatics Heinrich Heine University Düsseldorf Düsseldorf Germany; ^3^ Wildlife Sciences University of Göttingen Göttingen Germany

**Keywords:** *Alauda arvensis*, conservation, crop diversity, feeding frequency, habitat selection, synergetic effects

## Abstract

Reduced food availability during chick raising is a major driver of farmland bird declines. For the Eurasian Skylark (*Alauda arvensis*), food availability is determined by various factors (i.e., arthropod abundance/diversity, accessibility of the vegetation, distance to foraging sites). In modern farmland, it is supposed to decrease over the breeding season due to less penetrable vegetation. We explored foraging habitat selection by chick‐raising Skylarks with a focus on the seasonal dynamics of habitat use and food availability. We investigated (i) habitat selection concerning prey biomass/diversity, vegetation cover, and distance to foraging sites, (ii) the overall and seasonal habitat use, and (iii) seasonal developments of foraging parameters (e.g., the feeding frequency) as indicators of food availability. We collected data on foraging habitats and foraging parameters of chick‐raising Skylark pairs at 51 nests from a Central European population in 2018 and 2019. Prey biomass/diversity and vegetation cover were measured for all habitats around 42 of these nests. As revealed by multivariate and compositional analyses, Skylarks mainly selected foraging habitats based on the proximity to nests. The most frequent habitats within home ranges could not be ranked according to an overall importance for foraging and their use partially changed over time. The feeding frequency increased throughout the breeding season, while other foraging parameters did not show significant changes. In contrast to our expectations, our data indicated therefore an increase, not a decrease in food availability in the late breeding season. This also implies that the way in which Skylarks used habitats was constantly suitable to raise offspring. We interpret this to be a consequence of the heterogeneous farmland composition of the study area that enabled Skylarks to establish a diverse home range and to benefit from the synergetic effects of neighboring habitat types. Thus, our findings provide support for the high importance of crop diversity in Skylark conservation.

## Graphical abstract

The Eurasian Skylark (*Alauda arvensis*) is thought to suffer from a decrease in food availability .in European farmland during the breeding season. In contrast to this, our multivariate analyses of habitat selection and foraging parameters of chick‐raising pairs in Central Europe suggest no seasonal food shortage. We interpret our findings as being a consequence of the heterogeneous composition of our study area and highlight the importance of crop diversity in Skylark conservation.

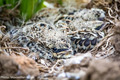

## 1 INTRODUCTION

Over the last 50 years, agricultural intensification in Europe has negatively affected the living conditions of numerous farmland‐associated birds, leading to severe population declines (Donald et al., 2006; Emmerson et al., 2016; Krebs et al., 1999). A primary driver behind these declines is reduced food availability together with a loss of suitable nesting habitats (Butler et al., 2007). Food availability, and therefore the ability to feed chicks, does not only depend on the sheer abundance of food but also on the accessibility and the distance to food sources, as in the case of the Eurasian Skylark (*Alauda arvensis*) (Jeromin, 2002; Wilson, 2001). Even though this species is still widespread across European agricultural land, its population in Europe has decreased by 54% since 1980 (Hagist & Zellweger‐Fischer, 2020; PECBMS, 2021).

Like many other songbirds (O'Connor, 1984), Skylarks mainly feed arthropods to their chicks (Poulsen et al., 1998; Weibel, 1999) and a diverse invertebrate diet is beneficial for chick development (Donald, Muirhead, et al., 2001). Plant‐based alternatives, even though regular components of the diet of Skylark nestlings (Ottens et al., 2014), are believed to be inferior food due to their poorer nutritional value (Douglas et al., 2012; Ricklefs, 1983). However, modern pesticides reduce the number and diversity of prey items either directly by killing insect pests together with collateral species or indirectly by killing undesirable weeds which are a food resource of many arthropods (Boatman et al., 2004; Hallmann et al., 2014; Odderskær, Prang, Elmegaard, et al., 1997). Furthermore, arthropod‐rich habitats, like fallow land, have strongly decreased in the European Union (EU) over the last decades (Tarjuelo et al., 2020). Besides the reduction of arthropod abundance and diversity, food accessibility can be lowered by unfavorable vegetation structure. Skylarks are passerines that collect food directly from the ground or near‐ground plant parts and thus depend on open vegetation that does not hamper mobility (Jenny, 1990a; Pätzold, 1983). However, many crops in modern agriculture become too dense during the breeding season of Skylarks, resulting in a decreasing amount of area that is available for foraging (Donald, 2004; Jenny, 1990a; Weibel, 1998). Especially Skylarks that settle in winter cereals are thought to suffer increasing food shortage later in the breeding season due to the growing sward structure (Donald & Morris, 2005). Therefore, conservation measures that prolong the access to food within winter cereals by implementing undrilled patches result in higher breeding productivity and better nestling condition (Morris et al., 2004). At the same time when the accessibility to foraging habitats decreases, the area of available breeding ground is highly reduced because Skylarks also build their nests on the ground in sparse vegetation (Donald, 2004; Jenny, 1990b). Thus, it is still unclear whether the lack of suitable nesting sites or the lack of suitable foraging sites explains the seasonal fall in territory density in winter cereals (Donald, 2004). Reduced food availability in farmland is further caused by landscape homogenization with an increase in field size and a decrease in crop diversity (Benton et al., 2003). These developments greatly limit the choice of foraging habitats, because Skylarks rarely fly more than 300 m between their nest and a foraging site (Jeromin, 2002; Wilson, 2001).

Consequently, analyses of habitat selection by chick‐raising Skylarks based on food availability should consider prey abundance and diversity, accessibility of vegetation, and the distance to a foraging site. Moreover, temporal effects on food availability should be included because arthropod abundance and vegetation structure per habitat type might change throughout the breeding season (Donald & Morris, 2005; Jenny, 1990a; Kuiper et al., 2013; Morris et al., 2004). Several researchers previously investigated foraging habitats of chick‐raising Skylarks and considered some of the influential parameters in various combinations (Jenny, 1990a; Jeromin, 2002; Kuiper et al., 2013; Murray, 2004; Weibel, 1998; Wilson, 2001). To our knowledge, however, studies that take into account all the mentioned determinants of food availability and measure their relative importance for habitat selection are still missing. Additionally, changes in habitat use over time have rarely been considered on a continuous scale, even though time‐scale dependencies are crucial for a better understanding of habitat selection (Miguet et al., 2013). Based on all the above, our study aimed to analyze the selection of foraging habitats by Skylarks in our Central European study area with a special focus on temporal dynamics. Furthermore, we aimed to find indications of a lowered food availability later in the breeding season due to grown vegetation that limited the access to food.

We divided our study into three parts. First, we analyzed the habitat selection of Skylarks with respect to arthropod abundance, insect diversity, vegetation structure, and distance to foraging sites and measured their relative importance for habitat choice. Second, we investigated both the overall and the seasonal use of different habitat types and interpreted it against the background of detected preferences from the step before. Finally, we checked if the ability of Skylarks to feed chicks decreased over time as a consequence of denser vegetation restricting the access to prey. We thus analyzed three foraging parameters as indicators of food availability. In a scenario with a decreasing amount of area that is available for foraging, we expected: (i) the feeding frequency to decrease because feeding Skylarks would need more time to find sufficient food. Furthermore, we expected both (ii) the distance flown to foraging sites and (iii) the actual area searched for food to increase throughout the breeding season to compensate for the overall loss of suitable foraging habitats.

## 2 METHODS

### 2.1 Study area

**FIGURE 1:**
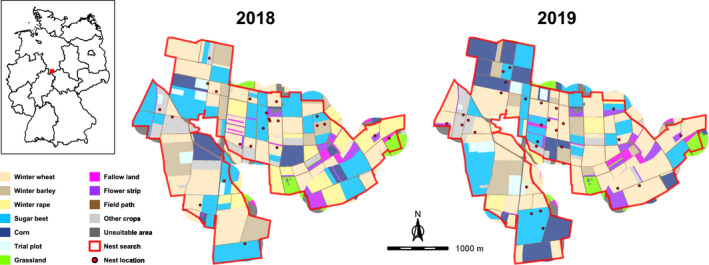
Location of the study area (red dot) within Germany (upper left corner) and its composition in 2018 and 2019. Other crops = asparagus, broad bean, clover, cup plant, potato, strawberry, summer barley, summer wheat, winter rye, and winter triticale; unsuitable area = building, company site, highway, water body, and wood. Only the area within the red line was searched for Skylark nests; arthropod and vegetation data were also collected outside the red line. Nest locations represent those nests with documented foraging flights. Shapefiles of individual fields were provided by the *Servicezentrum Landentwicklung und Agrarförderung*, shapefiles of Germany and its federal states by DIVA‐GIS (2021)

Fieldwork was conducted in the farmland south of the city Göttingen in Lower Saxony, Germany (N51°29.650, E9°56.635). Located in the transitional zone from maritime to continental climate of temperate latitudes, the area around Göttingen is relatively dry (mean annual temperature: 8.7°C, mean annual total precipitation: 644.9 mm) compared to other regions in Germany (Vohl, 2020). In the approx. 8.2 km² study site, the proportion of cropland (82.9%) outweighed the proportion of grassland (2.6%). Organic farming was practiced in 3.7% of the area. The average arable field size was 5.1 ha. Among the cultivated crops in 2018 and 2019, winter wheat (33.8% of the whole study site averaged over both years), sugar beet (19.9%), corn (9.0%), winter barley (7.8%), and winter rape (7.0%) were dominating. Other crops such as asparagus, broad bean, clover, strawberry, and summer wheat covered no more than 1.3% in each case. Moreover, the Faculty of Agricultural Sciences from the University of Göttingen cultivated 2.3% with trial plots of various crops. Sown flower strips (3.0%) together with fallow land (1.4%) were predominantly present in the eastern part. There, our study area partly intersected with a demonstration site of the Interreg North Sea Region project PARTRIDGE, which aims to increase biodiversity by establishing flower strips (PARTRIDGE, 2021). Field paths summed up to a network with a total length of ca. 32.8 km. Overall, the composition of the study area was heterogeneous without vast areas of monocultures (Figure 1). The estimated density of Skylarks at the study area was three to four territories per 10 ha (based on Langer 2017 and Meineke 2018, unpublished data).

### 2.2 Data collection

#### 2.2.1 Bird data

From April to August in 2018 and 2019, we searched for Skylark nests in our study area by observing Skylarks that clearly showed breeding behavior. The observation of adults carrying nesting material or prey items and of females returning to their nest for incubation were the main indicators to find the nests. In about one‐quarter of findings, rope dragging to flush incubating females supported the search. Moreover, the nest localization itself was facilitated by the use of a thermal binocular (Pulsar Accolade XQ38) in individual cases.

After a nest was found, nest content was checked on average every third day. In the case of nests with chicks, we used the state of physical development for aging as described in Pätzold (1983). Nest outcome was usually obvious, that is, predation could be confirmed due to injured / dead chicks or messy nesting material, while success could be confirmed by observing cheeping chicks in the nest surroundings or adults uttering warning calls when the nest was empty. Nests without a clear sign of outcome were interpreted as predated if chicks had not reached the age of the 7^th^ day, because Donald et al. (2002) found this to be the earliest age at which a nest was left successfully.

As a commonly used method for analyzing foraging habitats of chick‐raising Skylarks and other farmland birds (e.g., Douglas et al., 2009; Fischer et al., 2009; Kuiper et al., 2013), we directly observed foraging flights of feeding adults. When a Skylark returned to its nest with prey, the subsequent foraging flight was tracked with binoculars (8–10× magnification) until the bird landed. We documented the landing position on a map together with the habitat at that point and then directly focused observations on the nest again, waiting for the next foraging flight to start. A bamboo stick placed at a few meters distance to the nest helped the observer to visually locate it. In general, we carried out one observation session per nest with chicks per day. Each single observation session lasted until 10 foraging flights were recorded, up to a maximum of 90 min. The first observation session of a nest started as soon as possible, that is, not later than the day after a nest with chicks was found or after the regular nest control revealed that the chicks had already hatched. The series of observation sessions per nest ended when we found the nest to be predated or left successfully during a nest control or when the observer noticed deviant behavior. This included no activity at the nest indicating predation or feeding adults not landing at the nest anymore, but in the nest surroundings, indicating success. The following nest control then confirmed the observer's impression. Observations took place from an average distance of approx. 150 m to the nest in a hide like a car or camouflaged tent with a full view of all potential foraging habitats. We conducted our observation sessions at varying times during daylight and under all weather conditions with good sight, only avoiding storm, heavy rainfall and the hottest hours of a day with low feeding activity. Temperature and wind speed during the observations were taken as weather indicators. Data on these two variables were retrospectively downloaded from the Climate Data Center of the *Deutscher Wetterdienst* (Deutscher Wetterdienst, 2021b). We used the data from a weather station located within our study area (national station code: 1691). Data gaps (June 9^th^ to June 17^th^ in 2018 and on May 2^nd^ in 2019) were filled with data from a weather station in ca. 28 km distance (national station code: 2925), as daily temperature and wind speed were highly correlated between the stations in both years (Pearson's correlation coefficient for temperature = .993 and for wind speed = .852).

#### 2.2.2 Arthropod data

In order to analyze the foraging habitats of Skylarks against the background of food availability, we needed detailed information on arthropod biomass and prey diversity for all different habitats within a home range. Following the explanations by Kuiper et al. (2013) and Morris et al. (2007), vacuum sampling was chosen as the most suitable sampling method to gather data on relevant prey for Skylarks. We sampled each agricultural field (crop cultivation, grassland) and each non‐cropped field (flower strip, fallow land) that was at least partly inside a 300‐m radius around nests with chicks, as almost all foraging flights of Skylarks occur within that radius (Jeromin, 2002; Wilson, 2001). Because we assumed a homogenous distribution of arthropods per habitat unit, all individual fields were sampled only once. Additionally, we took one sample per 300‐m radius from the field path vegetation and, if occurring, from extensive areas of stunted growth within a field. One sample consisted of vacuuming the vegetation at no more than knee height down to the ground twenty times along a transect with a 1‐m distance to the preceding touchdown of the suction tube. As arthropod abundance and diversity can differ between the field edge and the field center (Batáry et al., 2012), we kept at least 5‐m distance from the habitat edge whenever possible. The samples were taken with an *ecoVac* (EcoTech Umwelt‐Meßsysteme, ⌀ 14 cm suction tube in 2018) and modified leaf vacuums (Stihl, ⌀ 11 cm in 2018; Stihl, ⌀ 14.5 cm in 2019) between 12:00 and 18:00 under dry weather conditions. Sampling took place, on average, 2 days after the chicks had left the nest or the nest had been predated. We froze the arthropod samples at −20° C for several days and then cleaned them from soil and debris. During the following counting of arthropods per sample, we identified each insect specimen to order level by the usage of a binocular microscope. Next, the samples were dried in drying cabinets at 105°C for 65 h and subsequently weighted with a precision balance (Sartorius).

Besides mapping prey within the area around Skylark nests, we aimed to systematically monitor the development of arthropod biomass and insect diversity for the most important habitat types of Skylark home ranges. The first preliminary results in 2018 had indicated winter wheat, sugar beet, corn, annual flower strips, and field paths as the main habitats. Thus, we took arthropod samples as described above in four fields of each main crop per half of a month (on the 7^th^ and 23^rd^) between May and July in 2019. Similarly, we sampled annual flower strips and field path vegetation at four different sites. In general, sample sites were chosen at the greatest possible distance to each other to ensure spatial independence. Data on arthropod number, taxonomic order in the case of insects, and dry weight were collected with the same methodology that we used for habitats around nests.

#### 2.2.3 Vegetation data

We measured vegetation openness for each habitat within the 300‐m radius around nests that we had vacuum‐sampled. As a proxy for openness, we used fractional vegetation cover (hereafter abbreviated as FVC or vegetation cover), which represents the proportion of ground covered by the vertical projection of foliage (Chianucci et al., 2018). The choice of this proxy was based on the assumption that Skylarks do not only depend on open vegetation to walk on the ground, but also to land in a specific habitat in the first place, so that we needed an indicator that considered the vertical vegetation structure as a whole. We took photos from each field with a straight‐down perspective at chest height similar to the photos that are required to measure FVC with automated tools (Patrignani & Ochsner, 2015). However, we estimated the vegetation cover visually. The use of automated tools was deemed unsuitable for our study because they focus on green vegetation while ignoring, for instance, brownish cereals later in the season. Visual estimations of FVC were independently conducted by three people using intervals of 10% in the range between 0% and 100%, thus following the recommendations by Hahn and Scheuring (2003) for cover estimation. The mean value was then calculated for subsequent analyses. FVC for habitats with a vegetation height up to 5 cm was set to zero because we did not expect a hampering effect of vegetation very close to the ground level. Similarly, we set the vegetation cover of field paths to zero, as Skylarks usually landed on their open ground and then walked to the wayside vegetation to forage.

We also documented the changes in the FVC at all sites chosen to systematically monitor arthropods for the most important habitat types. We measured the vegetation cover at the same time when arthropod samples were taken, that is, in each half of a month (on the 7^th^ and 23^rd^) between May and July 2019.

### 2.3 Data analysis

#### 2.3.1 Dataset

We found 96 active nests (i.e., nests with at least one laid egg) during the breeding seasons of 2018 and 2019. Of these, 22 nests became inactive (predation: 16, abandonment: 4, destruction by agricultural practices: 1, failed hatching: 1) before a record of foraging flights could start, 15 nests had a nest surrounding that was not observable, for example, due to hills, and 8 nests had chicks that were close to leaving or already sitting outside the nest at find. For the remaining 51 nests (Figure 1), we collected 2243 landing points of foraging flights. However, because the exact landing point was ambiguous in 2.4% of the cases, we only used the 2190 safe landing points for further analyses. We collected arthropod and vegetation data within a 300‐m nest radius for 42 of the 51 nests with documented foraging flights.

All recorded nest locations and safe landing points were digitized in *ArcGIS* (version 10.3.1; Esri Inc. 1999–2015; WGS 84 / UTM zone 32N). For the digital map of the study area in 2018 and 2019, we used shapefiles of the agricultural fields provided by the *Servicezentrum Landentwicklung und Agrarförderung* and modified them manually (e.g., by adding field paths). All subsequent analyses were conducted in R (version 4.0.3, R Core Team, 2020).

#### 2.3.2 Habitat selection

##### Influence of prey biomass and diversity, vegetation structure, and foraging distance

To understand how Skylarks select foraging sites, we combined our collected data on foraging flights, arthropods, and vegetation structure in the surroundings of Skylark nests. As a first step, we had to define a home range accessible for chick‐raising Skylarks. Following Kuiper et al. (2013), we calculated the 95^th^ percentile of all recorded distances between a nest and the corresponding landing points of foraging flights in both study years. Distances were determined with equal weighting to nests. The circular area around a nest with the resulting length of 188 m as radius was then defined as the home range. For the 42 nests of which we had mapped the surroundings in detail, we created digital shapefiles of the home ranges and intersected all habitats within this radius with the associated data on vegetation structure, arthropod biomass, and arthropod abundance. As a measure of insect diversity, we calculated the Shannon Index per individual habitat. In cases where part of the data was missing (e.g., because cows on a pasture prevented arthropod sampling), we used the mean values of the same habitat type within the home range if present. Otherwise, we kept the data gap.

Our analysis of habitat selection was conducted following the approach and explanations of Filla et al. (2021), that is, for each digital home range, we drew 240 random pseudo‐absence points to reach the recommended number of 10,000 points for good model performance (Barbet‐Massin et al., 2012). Landing points of foraging flights within the home range (1779) and all pseudo‐absence points (10,080) were intersected with the corresponding habitat characteristics. Next, we analyzed the influence of vegetation cover, arthropod biomass, insect diversity and distance between the nest and the point location on habitat selection with a generalized additive mixed model (GAMM). As pointed out by Guisan et al. (2002), general additive models are well suited to study ecological data due to their capacity for modeling nonlinear relationships. The point type (documented landing point = success, pseudo‐absence point = failure) was used as a binary response, while the individual nest was included as a random effect. Between predictors, Pearson's correlation coefficient was smaller than |.3| in all cases so that we did not expect multicollinearity to severely affect the explanatory power (Dormann et al., 2013). We weighted nests equally and pseudo‐absence points obtained the same total weight as documented landing points (Barbet‐Massin et al., 2012). The relative importance of all model variables for habitat selection was then analyzed with the random permutation procedure by Thuiller et al. (2009), as described in Filla et al. (2021).

##### Overall and seasonal use of habitat types

Before we investigated the seasonal habitat use of chick‐raising Skylarks, we first examined whether certain habitat types are generally more important for foraging than others during a whole breeding season. Therefore, we analyzed how the overall use of habitats differed from their availability within home ranges (third‐order habitat selection, Johnson, 1980). Again, we defined the circular area around a nest with a radius of 188 m as the home range. Then, we calculated the proportion of habitat types within this area for each of the 51 nests with documented foraging flights. We used the weighted surface area instead of the mere proportion to adjust for distance‐dependent habitat selection following Kuiper et al. (2013). Next, we calculated the relative use of habitats per nest by subdividing the number of documented foraging flights to the respective habitat by the total number of observed landing points. Only landing points within the respective home range were included. A compositional analysis according to Aebischer et al. (1993) was conducted to test for significant deviation from random habitat use and to rank habitats according to their relative importance as foraging habitat. *p*‐values were obtained by randomization (Manly & Navarro Alberto, 2020) with 1000 iterations. Specific categories were created for habitats present in at least one‐third of all home ranges, that is, winter wheat, sugar beet, corn, annual flower strips, and field paths. All other habitats were jointly analyzed under the category “other.”

To analyze the habitat use in relation to the time of the breeding season, we used mixed effect logistic regression models (GLMMs). For each previously analyzed habitat category except “other,” landing points within the home ranges were grouped into two categories: The habitat of the landing point equals the habitat in focus (i.e., success) or the habitat of the landing point does not equal the habitat in focus (i.e., failure). This binary categorization was then taken as the dependent variable, while the day of observation (day one: April 25^th^ as our earliest documented hatching date) was used as a predictor and the individual nest as a random effect. Additionally, we adjusted for the year and for the varying availability by including the weighted surface area. Correlation coefficients of Pearson's correlations between predictors were smaller than |.5|, indicating no serious distortion of model estimation through multicollinearity (Dormann et al., 2013). Nests with no occurrence of the focal habitat within their home range were excluded from the analysis, while the remaining nests (winter wheat: 45, sugar beet: 39, corn: 19, annual flower strip: 30, field path: 49) were equally weighted per day of observation.

We also intended to explain changes in habitat use based on the preferences in prey biomass and diversity as well as in the vegetation structure that we had analyzed before. That is, we visualized the temporal pattern of arthropod biomass, insect diversity, and vegetation cover per focal habitat using the data from our systematic monitoring.

#### 2.3.3 Foraging parameters

Based on the 51 nests with documented foraging flights, we analyzed the development of three foraging parameters throughout the breeding season as indicators of food availability: the feeding frequency, the distance flown to a foraging site, and the actual area that Skylarks searched for food.

To calculate the feeding frequency, we divided the number of recorded landing points of foraging flights per observation session by the minutes of observation. Consequently, our feeding frequency represented only a minimum value because it did not consider nest visits by feeding Skylarks with subsequent behavior other than foraging (e.g., males that started a song flight after feeding). Only observation sessions were included that ended before sunset, as feeding activity ceased during dawn (personal observation). This resulted in the full exclusion of one nest. Feeding frequencies of another nest were not considered, because the number of fed offspring was unclear due to the unknown fate of several chicks that had disappeared (partial brood loss vs. chicks left the nest asynchronously). For the remaining 49 nests, we calculated both the feeding frequency per hour as well as the feeding frequency per hour and chick. Our analysis of the distance flown to foraging sites was based on the distances that we had calculated between the 51 nests and the corresponding landing points of foraging flights.

For the actual area used for foraging, we defined the minimum convex polygon for 95% (MCP95) of all documented foraging flights per nest. We only considered those nests with at least 20 data points (46 nests) in our analysis. From then on, we did not see an increase in the used area with the number of landing points after visual inspection of this relationship. The feeding frequency per hour, the feeding frequency per hour and chick, and the distance flown were modeled with linear mixed effect models (LMMs). As predictors, we included the day of observation, the chick age, the starting time of the observation, both the temperature as well as the wind speed during the observation, and the year in all three models. Because 12 of the 51 nests with documented foraging flights had one radio‐tagged parent (with a tag weighing ca. 3% of the bodyweight) due to a parallel running telemetry study, we additionally included the radio‐tagging (yes / no) as predictor. Two of these nests were subsequent breeding attempts of the same bird. All nests were equally weighted, and the individual nest was included as a random effect.

For the analysis of the MCP95 size, we used a linear regression model (LM) with the day of hatching, the average temperature and wind speed during the observations, the radio‐tagging, and the year as predictors. To account for the varying daytime when the observation sessions took place, we found that averaging the starting time of the observations would be biologically meaningless. Instead, we grouped data points that were part of the MCP95 into “early” (collected during an observation session that started before noon, 12:00) and “late” observations (collected during an observation session that started after noon). Then, we calculated the proportion of early observations per MCP95 as further predictor. After each modeling, we used residual plots to check for homoscedasticity and both histograms and Q‐Q plots to check for normality of residuals. Pearson's correlations had coefficients smaller than |.6| in all models, so that we did not expect a serious bias of model estimation due to multicollinearity (Dormann et al., 2013).

## 3 RESULTS

### 3.1 Habitat selection

#### 3.1.1 Influence of prey biomass and diversity, vegetation structure, and foraging distance

Our GAMM model revealed a statistically significant effect of the following predictors: vegetation cover, insect diversity, and distance on the habitat selection of chick‐raising Skylarks. Only the effect of arthropod biomass was statistically insignificant (Table 1).

**TABLE 1:** Summary of the generalized additive mixed model describing the selection of foraging habitats by chick‐raising Skylarks (*Alauda arvensis*) with vegetation cover, arthropod biomass, insect diversity, and distance as predictors and the individual nest as random effect

Variable	*edf*	Ref.*df*	χ^2^	*p*
Vegetation cover	1.001	1.001	74.208	<.001
Arthropod biomass	1.000	1.000	0.934	.334
Insect diversity	3.036	3.691	10.723	.017
Distance	3.702	4.595	579.760	<.001
Nest	13.535	41.000	20.345	.019

Note

The model was based on 1779 landing points of 42 nests and 10,080 pseudo‐absence points. Penalized regression splines with maximum likelihood estimators were used for parameter smoothing. The estimated degrees of freedom (*edf*), reference degrees of freedom (Ref.*df*), chi‐square test statistics (χ^2^), and *p*‐values (*p*) are given. The model explained 16.9% of the deviance.

**FIGURE 2:**
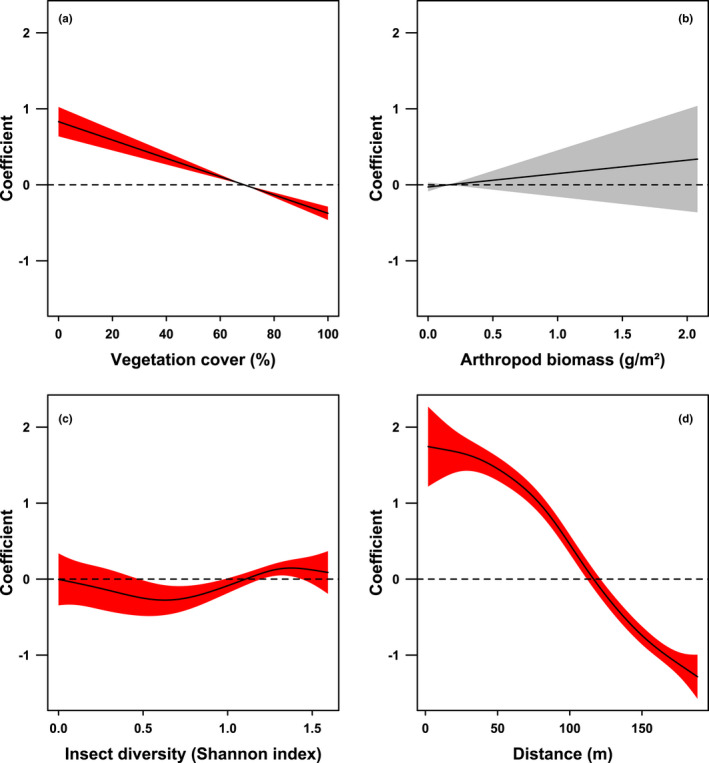
Selection of foraging habitats by chick‐raising Skylarks (*Alauda arvensis*) according to the generalized additive mixed model, which is based on 1779 landing points of 42 nests and 10,080 pseudo‐absence points. Penalized regression splines with maximum likelihood estimators were used for parameter smoothing. Plots show the selection (+95% CI) with respect to vegetation cover (a), arthropod biomass (b), insect diversity (c), and distance to the foraging habitat (d). Lower confidence intervals above the horizontal dashed line indicate statistically significant preference; upper confidence intervals below the dashed line indicate statistically significant avoidance. The confidence intervals of significant variables are red

Skylarks preferred a vegetation cover below 67% and avoided habitats with a cover above 70% (Figure 2a). While our results did not clearly point at habitat selection based on arthropod biomass (Figure 2b), Skylarks preferred habitats with a Shannon index between 1.2 and 1.4 and avoided habitats with a lower Shannon index, that is, between 0.5 and 1.0 (Figure 2c). Locations within a radius of 112 m around nests where preferred foraging habitats and locations outside a radius of 121 m around nests were avoided (Figure 2d).

According to our analysis of the relative variable importance, the distance between the nest and the habitat was clearly the dominating parameter influencing habitat selection (87.8%), followed by vegetation cover (9.9%). All other parameters had a relative importance below 1.5% (Table 2).

**TABLE 2:** Relative variable importance of the predictors (vegetation cover, arthropod biomass, insect diversity, distance) and the random effect (nest) in the generalized additive mixed model

Variable	Relative importance (%)
Vegetation cover	9.9
Arthropod biomass	0.1
Insect diversity	1.4
Distance	87.8
Nest	0.8

Note

The model describes the selection of foraging habitats by chick‐raising Skylarks (*Alauda arvensis*) based on 1779 landing points of 42 nests and 10,080 pseudo‐absence points.

#### 3.1.2 Overall and seasonal use of habitat types

**FIGURE 3:**
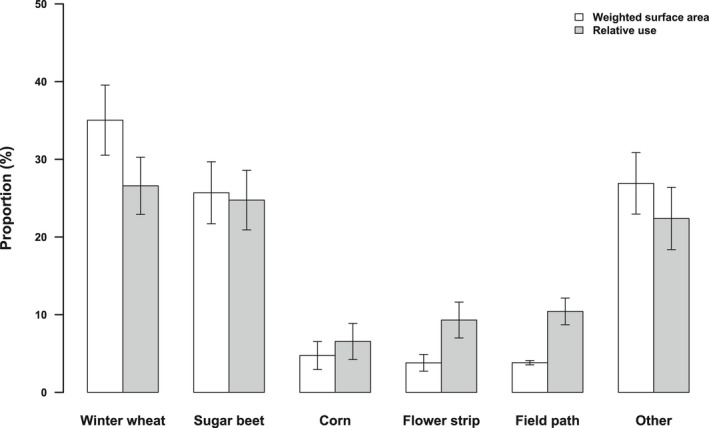
Average (+SE) weighted surface area compared to the average (+SE) relative use per habitat type and nest within home ranges of chick‐raising Skylarks (*Alauda arvensis*) over the whole breeding season. *n* = 51

Across all 51 nests with documented foraging flights, the average home range consisted to an extent of ca. 75% out of the 5 most frequent habitats, with ca. 35% winter wheat, 25% sugar beet, and roughly 5% corn, annual flower strips, and field paths in each case (Figure 3). About one‐quarter of foraging flights per nest was on average directed to both winter wheat and sugar beet, which therefore were not only the two most frequently available, but also the two most frequently used habitats. Approximately 10% of foraging flights were directed to both annual flower strips and field paths, clearly exceeding their respective availability. Further 7% of foraging flights per nest ended in corn, a use that is similar to its weighted surface area (Figure 3). Overall, within home ranges, habitat use of the whole breeding season did not differ significantly from random according to compositional analysis (Wilk's λ = 0.544, *p* = .119), making a ranking of the relative importance of habitats redundant.

**FIGURE 4:**
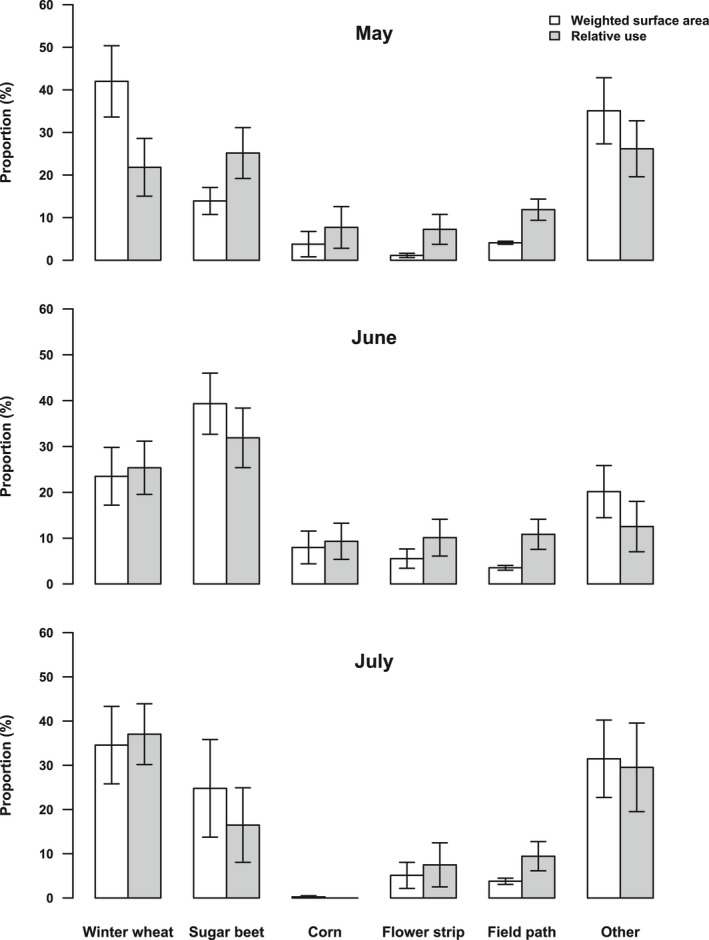
Monthly average (+SE) weighted surface area compared to the monthly average (+SE) relative use per habitat type and nest within home ranges of chick‐raising Skylarks (*Alauda arvensis*). The assignment of nest data to a month was based on the month of hatching. *n* (May) = 17; *n* (June) = 21; *n* (July) = 10. Data from nests whose chicks hatched during April are not shown due to the small sample size (*n* = 3)

When the data on habitat availability and use per nest were grouped based on the month of hatching, seasonal patterns became apparent (Figure 4). Changes in the average availability over time were a result of varying nest site locations of the nests we had found. Winter wheat and sugar beet were the most frequently used habitat types in all months, but the use of winter wheat in relation to its availability increased, while the use of sugar beet decreased. Likewise, annual flower strips were less used in relation to their availability later in the season due to an increasing proportion of the weighted surface area. The relation between the availability and use of field paths stayed constant. Corn was almost absent in the analyzed home ranges of Skylarks whose chicks hatched during July, resulting in a lack of use.

**FIGURE 5:**
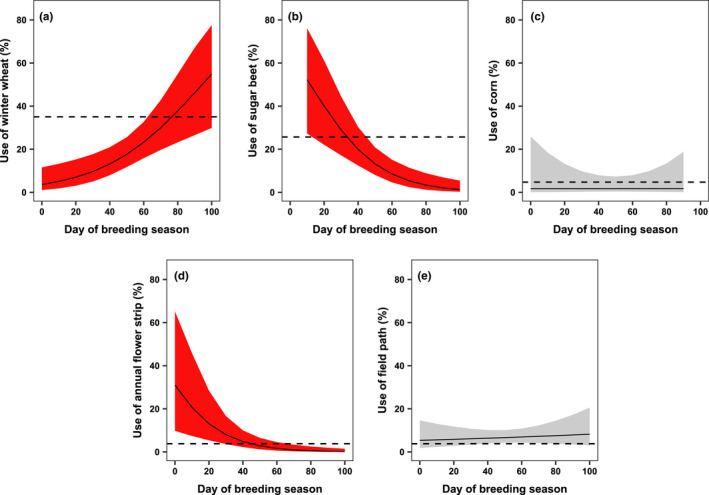
Relative habitat use (+95% CI) of winter wheat (a), sugar beet (b), corn (c), annual flower strips (d), and field paths (e) by chick‐raising Skylarks (*Alauda arvensis*) throughout the breeding season according to the predictions by mixed effect logistic regression models. A significant influence of the time is indicated by red confidence intervals. The predictions were made for the average weighted surface area, illustrated by the horizontal dashed line. Lower confidence intervals above the dashed line indicate statistically significant habitat preference; upper confidence intervals below the dashed line indicate statistically significant habitat avoidance. April 25^th^ was set as the 1^st^ day of the breeding season. Only data of home ranges were included where the respective habitat type was present. For sample sizes, see Table 3. Plots were created with the *ggemmeans* function of the R package *ggeffects* (Lüdecke, 2018)

The corresponding GLMMs in which we had adjusted for the weighted surface area detected statistically significant changes in the relative use of winter wheat, sugar beet, and annual flower strips in the course of the breeding season (Table 3, Figure 5a,b,d). Winter wheat was avoided as foraging habitat until the end of June and from then on used according to its availability. The predicted use of sugar beet matched almost the complete opposite time‐dependency with a use according to its availability until mid‐June and an avoidance afterward. Similarly, the selection of annual flower strips as foraging habitat decreased over time. The model predicted a preference until mid‐May, use according to their availability until the end of June and then avoidance until the end of the breeding season. The models of corn and field paths did not show a statistically significant effect of the day of the breeding season (Table 3, Figure 5c,e). Except for field paths, increasing habitat availability led to an increased use with statistical significance in all habitat models. Annual flower strips were significantly less used in 2019 compared to 2018 (Table 3).

**TABLE 3:** Summary of the mixed effect logistic regression models describing the relative use of habitats by chick‐raising Skylarks (*Alauda arvensis*) depending on the time of the breeding season

Model	Sample size	Fixed effect	Est.	SE	*z*	*p*
Winter wheat	1830 landing points of 45 nests	Intercept	−4.850	0.838	−5.791	<.001
		Day of breeding season	0.035	0.011	3.251	.001
		Weighted surface area (%)	0.045	0.008	5.361	<.001
		Year: 2019	−0.032	0.458	−0.070	.944
Sugar beet	1566 landing points of 39 nests	Intercept	−0.965	0.616	−1.568	.117
		Day of breeding season	−0.049	0.013	−3.752	<.001
		Weighted surface area (%)	0.069	0.010	6.553	<.001
		Year: 2019	−0.432	0.474	−0.913	.361
Corn	742 landing points of 19 nests	Intercept	−5.480	1.664	−3.293	.001
		Day of breeding season	0.000	0.027	0.010	.992
		Weighted surface area (%)	0.070	0.030	2.329	.020
		Year: 2019	2.172	1.327	1.637	.102
Annual flower strip	1160 landing points of 30 nests	Intercept	−0.934	0.774	−1.207	.228
		Day of breeding season	−0.055	0.016	−3.455	.001
		Weighted surface area (%)	0.198	0.039	5.033	<.001
		Year: 2019	−1.232	0.627	−1.965	.049
Field path	2000 landing points of 49 nests	Intercept	−3.012	0.815	−3.696	<.001
		Day of breeding season	0.005	0.010	0.454	.649
		Weighted surface area (%)	0.031	0.129	0.238	.812
		Year: 2019	0.050	0.466	0.108	.914

Note

The sample sizes, estimates (Est.), standard errors (SE), *z*‐values (z), and *p*‐values (*p*) are given for each model.

**FIGURE 6:**
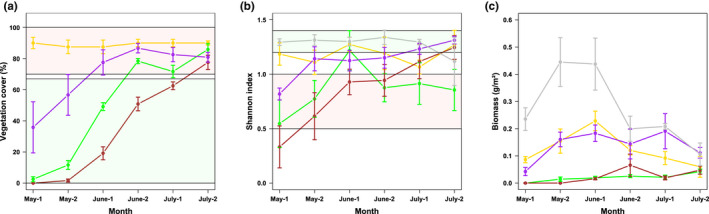
Average (+SE) vegetation cover (a), Shannon index (b), and arthropod biomass (c) of the main habitats within Skylark (*Alauda arvensis*) home ranges during the breeding season. Yellow line = winter wheat, green line = sugar beet, brown line = corn, purple line = annual flower strip, grey line = field path. Month‐1 = 7^th^; Month‐2 = 23^rd^. *n* (per habitat and half of month) = 4. Green boxes indicate the range that chick‐raising Skylarks preferred during foraging, red boxes indicate the range of avoidance. Vegetation cover of field paths was not visualized, as it had been defined as zero (see Methods)

Almost all analyzed habitats were within the preferred range of either vegetation cover or insect diversity for a certain time period (see section 3.1.1.), but not within both at the same time (Figure 6a‐b). Throughout the breeding season, the vegetation cover of winter wheat was ca. 90% and thus always within the range that Skylarks avoided during foraging (70%–100%). The vegetation cover of annual flower strips exceeded the preferred range, that is, cover below 67%, during early June, the vegetation cover of sugar beet during Mid‐June and of corn during Mid‐July. The Shannon index of sugar beet was smaller than 1.0 except during the first half of June and therefore within the range that Skylarks avoided (0.5–1.0), while the Shannon index of both winter wheat and annual flower strips was greater than 1.0 during most of the breeding season. Corn showed a steady increase in insect diversity with a Shannon index greater than 1.0 from the end of June onwards. The Shannon index of field paths was ca. 1.3 until mid‐July, which represented the highest insect diversity of monitored habitats and was within the range of Shannon indices that Skylarks preferred during foraging (1.2–1.4). Field paths also had the highest arthropod biomass until mid‐July (≥0.20 g/m²), followed by winter wheat and corn with intermediate biomass (0.05–0.20 g/m²) and sugar beet and corn with low biomass (≤0.05 g/m²) (Figure 6c).

### 3.2 Foraging parameters

**FIGURE 7:**
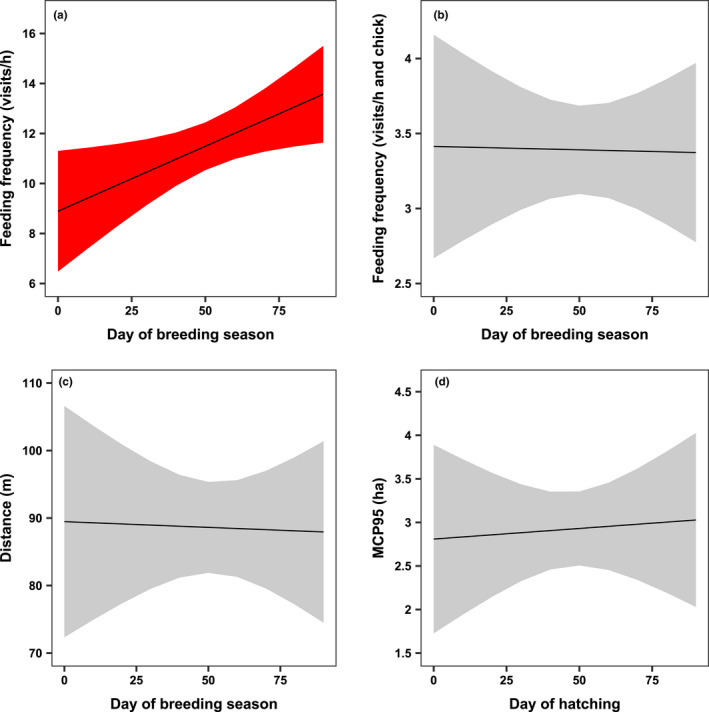
Development (+95% CI) of the foraging parameters feeding frequency per hour (a), feeding frequency per hour and chick (b), distance flown (c) and area searched for food (d) of chick‐raising Skylarks (*Alauda arvensis*) throughout the breeding season according to predictions by linear mixed effect models (a ‐ c) and a linear regression model (d). April 25^th^ was set as the 1^st^ day of the breeding season. A significant influence of the time is indicated by the red confidence interval. For sample sizes, see Table 4. Plots were created with the *ggeffect* function of the R package gg *effects* (Lüdecke, 2018)

Skylarks in our study area fed their chicks with an average frequency of 11.33 visits (±5.31 standard deviation) per hour and with an average frequency of 3.43 ± 1.60 visits per hour and chick. The feeding frequency per hour increased with statistical significance throughout the breeding season. In contrast, the feeding frequency per hour and chick did not change over time. Older chicks led to a statistically significant increase in both the feeding frequency per hour as well as the feeding frequency per hour and chick (Table 4, Figure 7a,b).

**TABLE 4:** Summary of the linear mixed effect models and the linear model describing the effects that influence the foraging parameters of chick‐raising Skylarks (*Alauda arvensis*)

Model	Sample size	Fixed effect	Est.	SE	*df*	*t*	*p*
Feeding frequency (visits/h)	221 feeding frequencies of 49 nests	Intercept	6.421	2.282	178.084	2.814	.005
		Day of breeding season	0.052	0.022	46.463	2.362	.022
		Chick age (days)	1.042	0.179	211.364	5.825	<.001
		Daytime (min)	−0.002	0.002	208.713	−1.309	.192
		Temperature (°C)	−0.101	0.076	206.231	−1.331	.185
		Wind (km/h)	−0.039	0.054	208.646	−0726	.469
		Radio‐tagging: yes	−1.139	1.173	50.567	−0.971	.336
		Year: 2019	−0.631	0.994	46.912	−0.634	.529
Feeding frequency (visits/h and chick)	221 feeding frequencies of 49 nests	Intercept	2.100	0.667	174.291	3.148	.002
		Day of breeding season	0.000	0.007	47.451	−0.067	.947
		Chick age (days)	0.331	0.051	209.065	6.460	<.001
		Daytime (min)	−0.001	0.000	205.328	−1.160	.248
		Temperature (°C)	−0.029	0.022	211.100	−1.336	.183
		Wind (km/h)	−0.011	0.015	205.404	−0.699	.486
		Radio‐tagging: yes	0.056	0.360	53.242	0.154	.878
		Year: 2019	0.281	0.307	47.758	0.915	.365
Distance flown to foraging habitat (m)	2,190 distances of 51 nests	Intercept	85.670	12.521	190.473	6.842	<.001
		Day of breeding season	−0.017	0.155	46.820	−0.109	.913
		Chick age (days)	1.898	0.748	1736.592	2.537	.011
		Daytime (min)	−0.008	0.005	2131.123	−1.548	.122
		Temperature (°C)	0.719	0.309	1639.687	2.322	.020
		Wind (km/h)	0.075	0.212	2128.517	0.355	.722
		Radio‐tagging: yes	−2.664	7.620	68.677	−0.350	.728
		Year: 2019	−32.321	6.939	47.032	−4.658	<.001
Area searched for food (ha)	46 MCPs	Intercept	6.365	1.893	–	3.363	.002
		Day of hatching	0.002	0.010	–	0.233	.817
		Observations before noon (%)	0.002	0.008	–	0.253	.802
		Mean temperature (°C)	−0.038	0.072	–	−0.527	.601
		Mean wind (km/h)	−0.096	0.071	–	−1.360	.182
		Radio‐tagging: yes	−0.608	0.534	–	−1.140	.261
		Year: 2019	−2.704	0.490	–	−5.522	<.001

Note

The sample sizes, estimates (Est.), standard errors (SE), degrees of freedom (*df*) in the case of the linear mixed effect models, *t*‐values (*t*), and *p*‐values (*p*) are given for each model.

Neither the distance flown, nor the area searched for food was affected by the ongoing breeding season, but instead by other predictors included in the respective model (Table 4, Figure 7c,d). The average distance flown to a foraging habitat was 86.17 ± 53.82 m. Distances significantly increased with chick age and temperature. Moreover, Skylarks flew significantly shorter distances in 2019 compared to 2018. The area that was actually used for foraging had a size of 2.92 ± 1.88 ha and was significantly smaller in 2019 than in 2018.

None of the four models showed a statistically significant effect of the radio‐tagging (Table 4).

## 4 DISCUSSION

### 4.1 Habitat selection

#### 4.1.1 Influence of prey biomass and diversity, vegetation structure, and foraging distance

Skylarks that search for food for their chicks have to consider the amount of prey, the accessibility of the vegetation, and the distance to a site when selecting a foraging habitat (Jeromin, 2002). In our study area, Skylarks based their selection mainly on two factors (Tables 1 and 2, Figure 2): the distance from the nest to the foraging habitat (87.8% relative importance) and the vegetation cover (9.9%).

The clear preference of foraging habitats closer than 112 m to the nest and avoidance of habitats beyond is in agreement with several other studies. For example, Kuiper et al. (2013) found an almost identical threshold for chick‐raising Skylarks in the Netherlands, where arthropod‐rich field margins were rarely used with distances to the nest above 100 m, while they were the preferred foraging habitat below that distance. Likewise, average flight distances recorded by Donald, Muirhead, et al. (2001), Jeromin (2002), and Murray (2004) were all beneath 100 m, although Poulsen (1996) found considerably higher distances averaging between 120 m and 230 m. As the flown distance is related to costs in time and energy (Poulsen, 1996), long extra‐territorial foraging flights are interpreted as signs of food shortage or the exploitation of very profitable food sources (Jenny, 1990a; Jeromin, 2002). Additionally, longer distances automatically reduce the time for birds to guard their nest, so that nest guarding is thought to be one positive side effect of the short flight distances by female Skylarks (Jeromin, 2002). Because distances above approx. 120 m were avoided, our results also indicate that measurements improving foraging habitat quality can only be successful if they are evenly implemented across landscapes. Supporting this, Kuiper et al. (2013) showed for their study area that breeding ground from where Skylarks reach field margins with short flights could have been almost doubled if field margins were implemented more systematically.

Vegetation cover was the second most important factor determining foraging habitat selection. Based on our results, Skylarks preferred vegetation cover below 70% for foraging and avoided higher FVC. The preference for sparse vegetation is also confirmed by several other studies and is strongly related to the foraging behavior of Skylarks (Jenny, 1990a; Murray, 2004; Odderskær, Prang, Poulsen, et al., 1997; Wilson, 2001). As they search for their prey on the ground during walking, Skylarks rely on vegetation that does not hamper mobility (Donald, 2004; Jenny, 1990a).

We found that insect diversity (1.4%) and arthropod biomass (0.1%) had the lowest relative influence on foraging habitat selection by Skylarks, with biomass being the only predictor without a statistically significant effect. The fact that Skylarks preferred habitats with higher insect diversity (Shannon index between 1.2 and 1.4) and avoided less diverse habitats (Shannon index between 0.5 and 1.0) can be explained by the beneficial effects of a varied diet on chick growth and condition (Borg & Toft, 2000; Donald, Muirhead, et al., 2001). Even though we did not detect an influence of arthropod biomass on habitat selection (in accordance with Murray, 2004), it is evident that a sufficient amount of arthropods is needed for breeding Skylarks. Several studies proved the negative impact of insecticides on insectivorous bird populations, including the Skylark (Hallmann et al., 2014; Odderskær, Prang, Elmegaard, et al., 1997). Instead, we think that chick‐raising Skylarks must consider first and most importantly the energetic costs of their foraging flight, and second arthropod reachability in terms of open vegetation, before they can profit from diverse and abundant prey items. This is also supported by Odderskær, Prang, Poulsen, et al. (1997) who frequently recorded foraging Skylarks on unvegetated tramlines later in the season, although arthropod abundance was higher within the dense crop itself. Moreover, since short distances clearly were the single most important determinant of habitat selection in our study area, we believe there was sufficient food availability within the direct nest surroundings.

#### 4.1.2 Overall and seasonal use of habitat types

Ranking the five most common habitat types within Skylark home ranges (winter wheat, sugar beet, corn, annual flower strips, and field paths) according to their general importance as foraging habitat was not possible. The reason for this was that habitat use within home ranges over the whole breeding season did not differ significantly from random which contrasts other studies using compositional analysis (Fischer et al., 2009; Kuiper et al., 2013; Weibel, 1998). However, this result depended on the number of habitat categories we included. When post hoc not only considering habitats as own category that were present in one‐third of all home ranges, but one‐quarter (so that winter barley was also a category of its own), the difference became significant (Table A1 in Appendix 1). Nevertheless, the ranking of the five most frequent habitats mainly was not supported with statistical significance, that is, ranks were mostly interchangeable. To us, this reflects the difficulty to group them into overall suitable and unsuitable foraging habitats. Throughout the breeding season, habitats offered either open vegetation or a high insect diversity/arthropod biomass, but rarely both (Figure 6). Moreover, we detected significant changes in the use of habitats over time in our GLMM models with adjustments for availability (Table 3, Figure 5). Vegetation that became too dense is likely the reason for the decreasing use of sugar beet and annual flower strips. The time when they were used less than available from the middle / end of June onwards coincided with the time when vegetation cover exceeded 70%. Douglas et al. (2009) found a similar shift in the use of field margins due to less accessible vegetation. As extensively used structures are very common measures to support Skylarks and farmland birds in general (Fischer et al., 2009; Kuiper et al., 2013; Ottens et al., 2014; PARTRIDGE, 2021), these results emphasize the relevance of low seeding densities when implementing flower strips. Our analyses further revealed an increased use of winter wheat later in the breeding season. Inevitably, a reduced use of certain habitats leads to an increased use of others. However, we were surprised about the intensified use of winter wheat, as the average vegetation cover was always within the avoided range (Figure 6). Additionally, winter wheat was of minor importance or clearly avoided as a foraging habitat in various studies (Jenny, 1990a; Kuiper et al., 2013; Wilson, 2001), and it is the most common example of a habitat that becomes unsuitable for foraging due to the growing sward structure (e.g., Donald & Morris, 2005). The bare tramlines which are frequently used micro‐habitats within cereals (personal observation, Odderskær, Prang, Poulsen, et al., 1997) may have been sufficiently profitable in our study area to be more exploited later in the season. This also demonstrates the limits of our study, as the vegetation and arthropod data that we extrapolated to field level cannot grasp fine‐scale differences influencing habitat use. In general, we believe that changing habitat characteristics and therefore the varying use of specific habitats over time impede an overall ranking and stretches the importance to consider time‐dependencies in analyses of habitat use.

Another important factor influencing the use of habitats is the nest site selection within the study area itself (second‐order habitat selection, Johnson, 1980) due to the strong distance‐dependent habitat choice. At first glance, for example, the decreasing use of sugar beet over time for a given availability (Figure 5b) seems to contradict its status as the constantly most frequented foraging habitat together with winter wheat (Figure 4). However, this can be traced back to the increased availability of sugar beet within home ranges later in the breeding season that was especially pronounced in June, because higher availability automatically led to intensified use (Table 3). At the same time, the average weighted surface area of winter wheat within home ranges was much smaller in June compared to May. We are aware that time patterns in the availability of habitats strongly depend on the nesting sites of the nests we found. Nevertheless, we think that the increasing availability of sugar beet / decreasing availability of winter wheat reflects the seasonal shifting of nest locations from winter cereals to summer crops well known from other studies (Schläpfer, 1988). As the increased availability of sugar beet over time did not come along with a proportional increase in its use, we believe that a shift in the nesting site was not triggered by foraging habitat preferences but by demands on the nesting site itself. As with foraging habitats, Skylarks depend on open vegetation that allows free access to the nest (Donald, 2004; Jenny, 1990b). However, from late May onwards, winter cereal vegetation becomes too dense, and Skylarks are forced to breed close to the bare tramlines or switch to a different crop (Donald, 2004; Donald et al., 2002; Fischer et al., 2009). Because tramlines are high‐risk nesting sites that Skylarks try to avoid (Püttmanns et al., 2021), sugar beet with a lower vegetation cover was probably more suitable than winter wheat.

### 4.2 Foraging parameters and food availability

Skylarks in modern agriculture are thought to experience a food shortage later in the breeding season due to the growing vegetation that hampers access to prey (Donald, 2004; Jenny, 1990a; Weibel, 1998). In contrast to this and our hypotheses, we did not find indications of a seasonal decrease in food availability (Table 4, Figure 7). Instead, the feeding frequency per hour showed a significant increase during the breeding season, implying an even greater food availability later in the season. When we modeled the feeding frequency per hour and chick, we found that this did not change significantly over time. Evidently, Skylarks invested the surplus of food in greater clutches later in the season, an effect already reported in the literature (Donald, Muirhead, et al., 2001), which caused an increase in the feeding frequency per hour. The lack of significant changes in the distance flown and the area searched for food at least do not support a food shortage scenario over time. We are aware that we did not collect data on chick weight, and therefore we cannot provide direct evidence that the food availability in our study area was sufficient for a healthy condition of chicks. However, only 1.7% of chicks (3 of 178) likely died because of starvation, while other studies documented higher losses (Jenny, 1990b; Poulsen et al., 1998; Wilson et al., 1997). The average values that we found for all foraging parameters were very similar to those reported in Jeromin (2002), who worked at a study site that was managed for farmland bird conservation. Furthermore, we think that the foraging parameters we analyzed have the potential to detect more subtle differences in food availability than the measurement of chick weight, as Skylark parents might be able to bear the costs of food shortage by an increase in feeding effort (Bradbury et al., 2003).

Besides our analyses of time effects, we found no significant influence of radio transmitters on any foraging parameter. Consequently, our results support other studies that could not document a detrimental effect of low‐weight tagging on the behavior of the focal species (e.g., Hegemann, 2012; Hegemann et al., 2010). Nevertheless, the negative impact of additional weight might be complex and more subtle (Hegemann et al., 2013), which was not possible to test in the framework of this study. Therefore, researchers should always consider potential costs for the bird and bias in data collection when using transmitters (Barron et al., 2010).

As we found no signs of a food shortage later in the season, but indications of an increase in food availability and thus feeding ability, we interpret the way in which Skylarks used habitats throughout the breeding season as constantly suitable to raise offspring. Even though higher vegetation cover was probably the reason for the reduced use of sugar beet and annual flower strips over time relative to their availability, accessible prey was apparently still numerous enough. We do not believe, however, that our findings can be readily transferred to other regions but result from the heterogeneous composition of the study area. The farmland south of Göttingen was characterized by a spatial arrangement in which winter crops and summer crops were often cultivated in fields next to each other (Figure 1). The implementation of several large flower strips, especially in the eastern part of the study area (PARTRIDGE, 2021), further enriched habitat diversity. Therefore, almost all Skylarks were able to compose a home range via nest‐site selection that included several habitat types (Table A2 in Appendix 1). This in turn enabled the use of spatial synergetic effects (Miguet et al., 2013), that is, to combine the advantages and outweigh the disadvantages of neighboring habitats at a given time with the potential for flexible adaptations of habitat use to changing conditions. A balanced home range composition with habitat complementation therefore prevented any deterioration of foraging parameters from our point of view. It is important to note, though, that we collected our data in two years of extremely dry weather (Deutscher Wetterdienst, 2021a; Zscheischler & Fischer, 2020) and that we could not consider the—often detrimental—effects of heavy rainfall (Donald, Buckingham, et al., 2001). Future research is required to compare our results with data both from more homogeneous landscapes and from years with changing weather conditions to corroborate our interpretations.

## 5 CONCLUSIONS

The results of this study support the often discussed benefits of heterogeneous farmland (Eraud & Boutin, 2002; Jeromin, 2002; Miguet et al., 2013; Schläpfer, 1988) with respect to foraging habitats of Eurasian Skylarks. In contrast to most other studies that infer positive effects of crop diversity indirectly from analyzing abundance patterns (Hiron et al., 2012), we draw our conclusions based on direct observations of habitat use in combination with measurements of food availability. As we could show, Skylarks acted on comparatively small spatial scales, avoiding distances to food sources longer than 120 m. We suggest that spatial synergetic effects of different habitats within a home range secured sufficient food availability throughout the breeding season. Foraging habitats with vegetation densities below 70% are of special importance, an aspect that should be considered when implementing conservation measures such as flower strips.

We find it encouraging to see that it can be possible for chick‐raising Skylarks to find enough food throughout the breeding season even in conventionally managed farmland— under the premise of habitat heterogeneity.

## CONFLICT OF INTEREST

The authors declare that they have no conflict of interest.

## AUTHOR CONTRIBUTIONS

**Manuel Püttmanns:** Conceptualization (equal); Data curation (equal); Formal analysis (equal); Funding acquisition (equal); Investigation (equal); Methodology (equal); Project administration (lead); Visualization (equal); Writing – original draft (lead). **Laura Böttges:** Investigation (equal); Writing – review & editing (equal). **Tim Filla:** Formal analysis (equal); Methodology (equal); Writing – review & editing (equal). **Franziska Lehmann:** Data curation (equal); Investigation (equal); Writing – review & editing (equal). **Annika Sophie Martens:** Investigation (equal); Writing – review & editing (equal). **Friederike Siegel:** Investigation (equal); Writing – review & editing (equal). **Anna Sippel:** Investigation (equal); Writing – review & editing (equal). **Marlene von Bassi:** Investigation (equal); Writing – review & editing (equal). **Niko Balkenhol:** Conceptualization (equal); Supervision (equal); Writing – review & editing (equal). **Matthias Waltert:** Conceptualization (equal); Supervision (equal); Writing – review & editing (equal). **Eckhard Gottschalk:** Conceptualization (equal); Funding acquisition (equal); Investigation (equal); Methodology (equal); Supervision (lead); Writing – review & editing (equal).

## Data Availability

All datasets and R scripts used to generate the results presented in this study are available at the Dryad Digital Repository: https://doi.org/10.5061/dryad.bg79cnpc2.

## APPENDIX 1

**TABLE A1:** Compositional analysis matrix of the means of log‐ratio differences and ranking of the habitats that were available within one‐quarter of Skylark (*Alauda arvensis*) home ranges. The habitat use differed significantly from random (Wilk's λ = 0.320, *p* = .047). Numerator habitats are in rows; denominator habitats are in columns. Numbers in brackets represent the sample size to calculate the respective means of log‐ratio differences. **p* < .05

	Winter wheat	Field path	Annual flower strip	Sugar beet	Corn	Winter barley	Other	Rank
Winter wheat	0.000	0.766 [43]	0.538 [27]	1.301 [33]	2.943 (*) [15]	4.293 [10]	2.190 (*) [45]	**6**
Field path		0.000	0.411 [29]	1.307 [38]	2.764 [19]	3.654 (*) [14]	1.532 [47]	**5**
Annual flower strip			0.000	0.506 [21]	1.141 [12]	1.679 [7]	2.490 (*) [29]	**4**
Sugar beet				0.000	0.366 [16]	1.605 [10]	2.054 [37]	**3**
Corn					0.000	5.148 (*) [8]	1.632 [17]	**2**
Winter barley						0.000	0.181 [13]	**1**
Other							0.000	**0**

**TABLE A2:** Weighted surface area (%) of the main habitats within Skylark (*Alauda arvensis*) home ranges and the total number of different habitats per home range with a weighted surface area ≥1% (No. habitats). It is given the minimum number of different habitats, because the category “other” might have consisted of more than one habitat type

Nest	Winter wheat	Sugar beet	Corn	Annual flower strip	Field path	Other	No. habitats
N02.18	9.3	25.3	0.0	0.0	4.2	61.2	**4**
N03.18	75.9	3.1	5.4	3.9	2.7	9.0	**6**
N04.18	77.7	9.8	3.6	1.7	5.6	1.6	**6**
N05.18	57.2	27.6	0.0	8.1	4.7	2.4	**5**
N09.18	66.6	29.9	1.6	0.0	0.6	1.4	**4**
N10.18	0.0	32.1	0.0	0.0	2.7	65.2	**3**
N12.18	56.8	2.9	0.0	1.7	4.7	34.0	**5**
N13.18	0.0	15.5	0.0	0.1	2.6	81.9	**3**
N14.18	3.8	0.0	0.0	0.4	4.8	91.0	**3**
N16.18	53.8	26.8	0.0	0.0	8.1	11.3	**4**
N18.18	75.4	1.3	0.5	0.0	5.3	17.6	**4**
N20.18	12.0	69.9	0.0	0.0	0.0	18.1	**3**
N22.18	42.1	34.8	0.3	14.5	5.3	3.0	**5**
N23.18	4.3	20.0	0.0	2.6	3.6	69.4	**5**
N24.18	3.2	0.0	0.0	23.6	1.3	72.0	**4**
N25.18	8.8	56.7	2.4	22.0	7.5	2.6	**6**
N26.18	4.9	68.5	0.0	0.0	3.4	23.2	**4**
N27.18	21.6	62.6	0.0	0.0	5.3	10.5	**4**
N30.18	9.9	86.1	0.0	0.1	2.9	1.0	**4**
N32.18	81.2	3.0	0.0	9.2	3.6	2.9	**5**
N33.18	32.3	5.2	0.0	0.0	4.4	58.2	**4**
N35.18	54.1	0.6	0.0	12.8	5.8	26.7	**4**
N36.18	30.5	7.0	2.5	0.0	4.1	55.9	**5**
N02.19	85.0	0.0	0.0	1.8	3.7	9.6	**4**
N04.19	70.9	0.0	8.0	0.0	5.0	16.1	**4**
N05.19	78.4	0.0	0.0	5.5	3.9	12.2	**4**
N08.19	62.5	19.1	0.0	0.8	4.3	13.3	**4**
N09.19	77.4	0.0	0.0	0.0	4.7	17.9	**3**
N12.19	88.4	0.0	0.0	0.5	3.3	7.8	**3**
N17.19	52.5	32.2	0.0	0.0	5.8	9.6	**4**
N26.19	5.7	6.4	0.0	0.0	4.4	83.6	**4**
N27.19	82.7	0.0	0.2	0.3	7.0	9.8	**3**
N30.19	4.8	1.6	50.9	0.1	3.3	39.3	**5**
N31.19	0.0	29.9	2.2	2.0	6.3	59.7	**5**
N34.19	2.7	0.0	0.0	0.5	4.3	92.6	**3**
N35.19	54.2	5.8	0.0	16.7	4.3	19.0	**5**
N36.19	89.5	0.3	2.4	0.4	4.7	2.8	**4**
N37.19	0.0	61.2	30.4	0.1	5.5	2.8	**4**
N38.19	0.0	69.7	26.9	0.0	3.4	0.0	**3**
N40.19	65.7	0.0	27.4	0.2	0.8	5.9	**3**
N41.19	65.0	0.0	0.0	28.3	0.0	6.8	**3**
N43.19	10.7	88.0	0.0	0.0	1.3	0.0	**3**
N44.19	20.0	78.1	0.4	0.0	1.1	0.5	**3**
N45.19	0.0	60.5	8.4	0.0	1.3	29.9	**4**
N46.19	1.4	16.6	65.0	1.3	1.9	14.0	**6**
N47.19	10.8	57.7	0.0	0.0	1.7	29.8	**4**
N48.19	9.7	49.1	0.0	30.9	2.1	8.1	**5**
N51.19	0.3	68.1	3.5	3.2	3.8	21.1	**5**
N52.19	11.8	69.7	0.0	0.0	6.1	12.4	**4**
N54.19	53.8	0.0	0.0	0.0	0.6	45.6	**2**
N57.19	2.4	7.8	0.0	0.7	6.9	82.2	**4**
